# Microgravity induces proteomics changes involved in endoplasmic reticulum stress and mitochondrial protection

**DOI:** 10.1038/srep34091

**Published:** 2016-09-27

**Authors:** Bryan J. Feger, J. Will Thompson, Laura G. Dubois, Reddy P. Kommaddi, Matthew W. Foster, Rajashree Mishra, Sudha K. Shenoy, Yoichiro Shibata, Yared H. Kidane, M. Arthur Moseley, Lisa S. Carnell, Dawn E. Bowles

**Affiliations:** 1 Department of Surgery, Duke University Medical Center, Durham, NC 27710, USA; 2 Duke Proteomics and Metabolomics Shared Resource, Duke University Medical Center, Durham, NC 27710, USA; 3 Department of Medicine, Duke University Medical Center, Durham, NC 27710, USA; 4 Department of Genetics, the Carolina Center for Genome Sciences, and the Lineberger Comprehensive Cancer Center, University of North Carolina, Chapel Hill, NC 27599, USA; 5 Wyle Science, Technology and Engineering Group, Houston, TX 77058, USA; 6 NASA Johnson Space Center, Houston, TX 77058, USA; 7 NASA Langley Research Center, Hampton, VA, 23666, USA

## Abstract

On Earth, biological systems have evolved in response to environmental stressors, interactions dictated by physical forces that include gravity. The absence of gravity is an extreme stressor and the impact of its absence on biological systems is ill-defined. Astronauts who have spent extended time under conditions of minimal gravity (microgravity) experience an array of biological alterations, including perturbations in cardiovascular function. We hypothesized that physiological perturbations in cardiac function in microgravity may be a consequence of alterations in molecular and organellar dynamics within the cellular milieu of cardiomyocytes. We used a combination of mass spectrometry-based approaches to compare the relative abundance and turnover rates of 848 and 196 proteins, respectively, in rat neonatal cardiomyocytes exposed to simulated microgravity or normal gravity. Gene functional enrichment analysis of these data suggested that the protein content and function of the mitochondria, ribosomes, and endoplasmic reticulum were differentially modulated in microgravity. We confirmed experimentally that in microgravity protein synthesis was decreased while apoptosis, cell viability, and protein degradation were largely unaffected. These data support our conclusion that in microgravity cardiomyocytes attempt to maintain mitochondrial homeostasis at the expense of protein synthesis. The overall response to this stress may culminate in cardiac muscle atrophy.

Although all life on Earth has evolved in the presence of gravity, we are only beginning to appreciate the fundamental role that gravity plays in terrestrial biology. Because of our desire to travel off this planet, humans are beginning to place biological systems, themselves included, in environments of minimal gravity, or microgravity. Reports of decreased immune function, bone density loss, skeletal muscle atrophy, and effects on cardiac physiology of astronauts on off-planet missions indicate that microgravity is a cellular stressor that has the capability to detrimentally affect human health^1^,^2^. As we consider extended travel beyond Earth’s orbit, astronauts will be subjected to extreme temperature, oxidative stress, radiation exposure, and other environmental stress in addition to microgravity. Elucidating independent effects of microgravity on biological systems will help us assimilate potential additive or synergistic effects, confounded by known biological stressors. A logical extrapolation to comprehending the impact of microgravity on biological systems within an organism will require fundamental knowledge of how microgravity influences molecular processes within cellular compartments.

Our understanding of the molecular pathways involved in the response to microgravity has derived from studies of experiments performed during space flight, studies of astronauts, and modeling weightlessness through hind limb unloading of animal models or cell-based experiments in bioreactors^3^,^4^,^5^,^6^. These studies, focusing predominantly on bone, immune, nervous, and skeletal muscle cells, have revealed pleiotropic effects rendered by microgravity, such as altering cytoskeletal content, directing cells toward apoptotic death or premature senescence, altering cellular division and differentiation, and influencing protein turnover^7^,^8^. These changes are mediated through a myriad of possible biological pathways orchestrated by alterations of gene expression and signal transduction pathways^9^. Which stress-response proteins are activated in response to microgravity and the downstream consequences of their actions is poorly understood mechanistically.

To reveal outcomes of microgravity on molecular processes within the cellular environment, we have employed a mass spectrometry-based proteomics approach. Proteomics analysis based on mass spectrometry allows for the relative quantitation of a large number of proteins concurrently, and in a relatively unbiased manner^10^. Mass spectrometry-based proteomics can be rendered even more informative by addition of a labeling component to understand the dynamics of the changing protein content. Use of combinations of proteomics methodologies are powerful tools for examining an array of biological pathways and networks in a single experiment^11^,^12^. In this study, we utilized a combination of proteomics techniques, namely label-free quantification and dynamic stable-isotope labeling by amino acids in cell culture (Dynamic SILAC)^13^ to characterize the microgravity stress response in primary cardiomyocytes. Our results suggest that cardiomyocytes respond to the stress of microgravity by coordinating a reduction in protein synthesis while maintaining the integrity and function of mitochondria.

## Results

### Protein abundance is altered in cardiomyocytes during simulated microgravity

**Figure 1:**
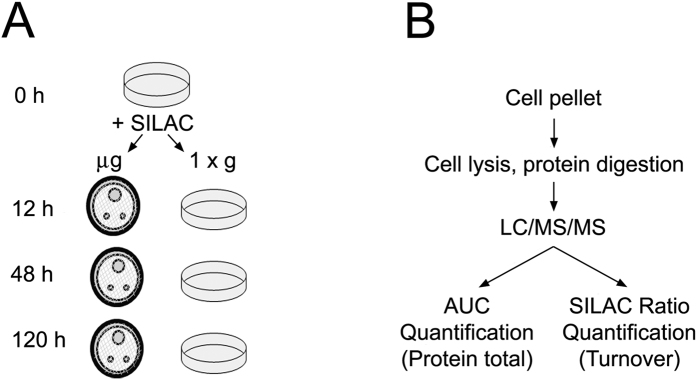
Overview of study work flow. (**A**) Schematic overview of SILAC experiments. NRCMs were placed in SILAC and split into groups designated for 12, 48, and 120 h following which cell pellets were analyzed. (**B**) Mass Spectrometry work flow. Cell pellets were lysed, proteins were digested and analyzed by area under the curve (AUC) following separation through LC/MS/MS.

A rotating wall bioreactor engineered by NASA was used to simulate microgravity^14^. This bioreactor perpetually suspends cells without inducing shear, thereby creating a net gravity vector of zero^3^,^15^. Primary rat neonatal cardiomyocytes were placed into simulated microgravity (*μg)* or normal gravity (*1xg*) conditions (Fig. 1A). High-resolution nanoscale liquid chromatography tandem mass spectrometry (LC-MS/MS) was performed as outlined in Fig. 1B on cells isolated over time at 12, 48, or 120 h from *μg* or *1xg* conditions.

**Figure 2:**
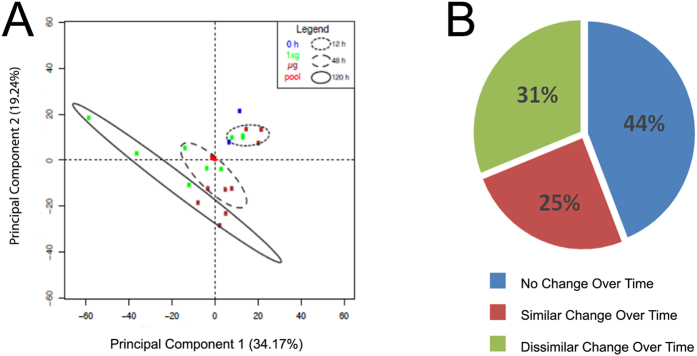
Protein abundance is altered in cardiomyocytes during simulated microgravity. (**A**) Principal components analysis (PCA) was performed using protein data from Supplemental Table S3, z-score normalized. The smallest variability among any sample group is among 0 h and 12 h samples, followed by 48 h and 120 h. The QC pool samples are nearly indistinguishable in the center of the PCA, showing technical variability is far less than biological variability. (**B**) Fraction of proteins that changed as a function of time or between groups.

Intensity values for 6,174 peptides (Supplemental Table 2; peptide expression) were utilized to measure the relative expression for 848 proteins across all time points (0, 12, 48, and 120 h) and gravity conditions (*μg* or *1xg*) (Supplemental Table 3; protein expression). Principal components analysis (PCA) demonstrated that as a whole, protein abundance between *μg* and *1xg* samples were not different at 12 h, only slightly different at 48 h, and highly different at 120 h (Fig. 2A).

ANOVA was performed on log 2-transformed data to evaluate the relative abundance of individual proteins as a function of gravity condition at each time point, and longitudinally as a function of time under each gravity condition (Supplemental Table 3-Protein expression). From these analyses, proteins were classified into three groups (Fig. 2B): (1) no change over time under *1xg* or *μg* (44%); (2) similar change over time or temporal protein trend under *1xg* or *μg* (25%); and 3) dissimilar change over time between *1xg* or *μg* (31%).

### Cardiomyocytes under simulated microgravity have diminished protein turnover

In addition to the unbiased proteomics that measures relative abundance of protein, we included the technique of dynamic stable-isotope labeling in cell culture (dynamic SILAC) (Fig. 1A) to measure protein turnover. Media containing heavy isotope versions of arginine and lysine was added to cells at the inception of the experiment. At each time point, LC-MS/MS peak intensities were then examined to derive the light (I_L_) and heavy (I_H_) isotope-derived peaks based on accurate mass and retention time (Supplemental Fig. 1). The intensities of I_L_ and I_H_ versions of each quantified peptide were summed to yield a total peptide intensity using Equation 1 (Supplemental Fig. 2). To estimate protein turnover, we utilized the ratio of I_H_ peptide to the total of I_L_ and I_H_ — the relative isotope abundance (RIA) — following stable-isotope incorporation into newly-synthesized protein using Equation 2 (Supplemental Fig. 2).

Multiple scenarios exist to explain the distribution of I_L_ and I_H_ over time, and four major possibilities are shown in Supplemental Table 1. At the initial time point of label addition, a peptide would have no stable-isotope incorporation (I_H_ = 0; Supplemental Fig. 1A), and thus I_L_ would be equal to the total amount of peptide. In Supplemental Fig. 1B, there is the same theoretical quantity of total peptide (I_H_ + I_L_ = 1); however, half of the peptide has been generated since the time = 0, thus the peptide RIA is 0.5. States C and D show a 50% reduction in peptide abundance compared to States A and B, and also show RIA of 0.5 and 0.8, respectively (Supplemental Fig. 1C,D). In this manner, the same dataset can be used to measure both total protein differential expression based on standard label-free proteomics by summing the intensities of all forms of all peptides and relative turnover by calculating the RIA of individual peptides using the heavy and light forms.

**Figure 3:**
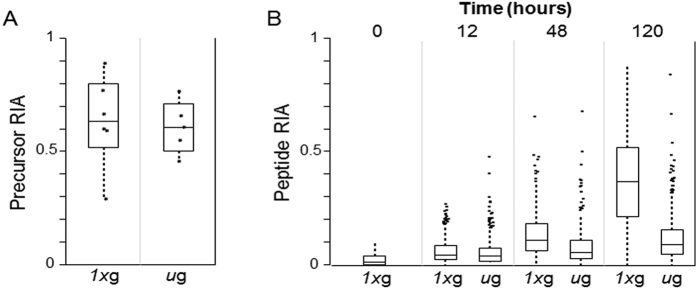
Protein turnover is diminished in microgravity. (**A**) A statistical box plot for the precursor amino acid ‘pool’ RIA calculated using the *I*_*H*_/*I*_*H*_ and *I*_*H*_/*I*_*L*_ forms of the double-lysine containing peptide EATNPPIIQEEKPK from Protein Disulfide Isomerase A3, including 48 h and 120 h time points. These data show no difference in the amino acid pool available for protein synthesis inside the cell between *1xg* and *μg* condition (*p* = 0.80, Students T-test). (**B**) Statistical boxplots for RIA calculated for 359 highly-curated peptide pairs as a function of time under either *1xg* or *μg* environment. Peptide RIA, a measure of protein turnover, was not different at 12 h (p = 0.32, Students T-test) but showed statistically significant slowing under *μg* at 48 and 120 h (p < 1e-6, Students T-test). Boxes represent quartiles.

Estimation of protein turnover requires the measurement of RIA of the precursor amino acid pool (essentially the maximum possible protein incorporation) by utilizing a peptide with more than one labeled residue^13^,^16^. We assessed precursor amino acid RIA (r) using the peptide EATNPPIIQEEKPK in our data set (Supplemental Table 2-Peptide Expression) by the method of Doherty *et al*.^16^. Since this peptide contains two lysine (K) residues, the relative abundance of the heavy/heavy and heavy/light forms is dependent only on the ratio of the heavy and light lysine isotopes at the time of protein synthesis and is independent of protein turnover. This global measurement, defined as precursor RIA, was performed on data collected at 48 h and 120 h for both *μg* and *1xg* (Fig. 3A). No significant difference in RIA was observed for the conditions (p = 0.80, Students T-test), indicating that the switch from light to heavy amino acid media was performed equally for both conditions. Additionally, since this is an intracellular measure of precursor amino acid, it indicates that there likely was no significant dysregulation in amino acid transporter function at the cell membrane between *μg* and *1xg*. Collectively, these data suggest that incorporation of precursor amino acid (at least K and R) into proteins occurs similarly under both normal and microgravity conditions.

Next, the RIA for 359 peptides passing strict selection criteria (see Methods, Table S4-Dynamic SILAC) was determined for 0, 12, 48, and 120 h under *μg* or *1xg* (Fig. 3B). At 12 h, there were no significant differences in protein turnover in *μg* versus *1xg*, as measured by RIA (p = 0.32, Students T-test). However, there were large differences in protein turnover at both 48 and 120 h in *μg* versus *1xg* (p < 1e^−6^ for both; Fig. 3B). These data indicate that the drastic decrease in protein turnover in simulated microgravity, despite there being no difference in the abundance of the intracellular precursor amino acid pool (Fig. 3A) and despite there being no difference in total expression levels of a majority (~70%) of the proteome during the microgravity exposure (Fig. 2B).

### Microgravity differentially influences the endoplasmic reticulum and mitochondrial proteome and stress pathways

The results from both the abundance and SILAC proteomics data sets indicated that there were a number of proteins significantly changed in abundance or turnover in response to simulated microgravity at 120 h. Each data set (abundance and turnover) was analyzed using a bioinformatics/gene functional-enrichment approach, as outlined in the materials and methods, to map these changes to cellular location, protein function, and biological pathways. Following individual analysis, a comparative analysis of abundance and turnover was performed with the aim of elucidating any underlying commonalities and differences in quantitative and dynamic protein expression patterns for exposure of the cardiomyocytes to microgravity.

Spectra from LC-MS/MS analyses (Supplemental Table 2) were assigned to peptides (and thus putative protein precursors) using a search against the NCBI RefSeq database^17^. The protein identifiers were mapped into the corresponding genes that encode for the proteins by using Bioconductor’s MyGene package^18^ in order to carry out the translation from protein space to gene space; this mapping was critical since most functional annotation data sets, including the Gene Ontology^19^, are based on gene identifiers as opposed to protein. The differentially abundant proteins in these data sets were determined using a combination of t-test and ANOVA analysis with a p-value cut-off ≤ 0.05 after multiple hypothesis testing correction using Benjamini and Hochberg procedure. After identification of significantly impacted proteins, a functional enrichment analysis was performed with the aim of identifying biological processes, cellular components, or molecular functions that are significantly impacted by microgravity. Using the transformed proteomics data and the gene functional annotations, a pathway enrichment analysis was performed using Gene Set Enrichment Analysis (GSEA) software independently on either the protein abundance (Table 1) or SILAC turnover (Table 2) data sets at 0.05 False Discovery Rate (FDR) cutoff. Relative to normal gravity, metabolic gene sets in microgravity were exclusively up-regulated in the protein abundance data, whereas gene sets related to cardiac/heart muscle contraction and development were uniquely up-regulated in the protein turnover data. Gene sets related to mitochondrial and electron-transport processes and gene sets annotated with Alzheimer’s, Huntington’s, and Parkinson’s diseases, neurological diseases originating in tissues with limited regenerative capacity similar to cardiac tissues, were up-regulated in both protein abundance and turnover data sets. Regarding down-regulated gene sets, the microgravity protein abundance data were enriched with gene sets associated with the ribosome, transcription and translation, whereas the protein turnover data were predominantly related to functions related to protein/macromolecule localization.

**Figure 4:**
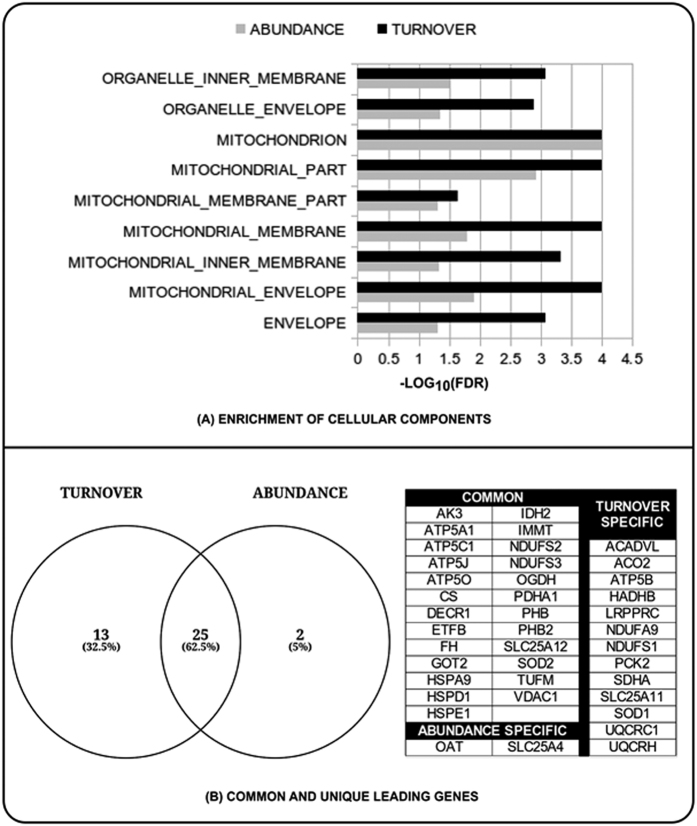
Gene Ontology. (**A**) Comparison of enrichment of Gene Ontology cellular components for protein abundance and turnover data sets. (**B**) Leading genes that contributed to the difference and similarity in the perturbation of the cellular components shown in panel A. A 0.05 FDR cutoff value was used for this analysis.

The analysis of the combination of abundance and turnover data revealed that the mitochondria was more impacted than other organelles in the cell (Fig. 4A). However, it appears that mitochondrial turnover was more impacted than protein abundance. A total of 40 proteins from both data sets mapped to these mitochondrial processes (Fig. 4B). Two proteins were unique to the abundance data set, 13 were unique to the turnover data, and 25 proteins were common to both data sets. We mapped these common genes to pathways using the Pathway Commons database (results not shown here). The top significant pathways include the citric acid cycle (TCA), ATP synthesis, nuclear estrogen receptor network, and metabolism of carbohydrates, amino acids, and glucose.

One limitation from the above analysis was that the data set subjected to enrichment analysis was restricted to proteins for which we had information on cellular location to organelles; this analysis was accomplished using data only from GO of which there were only 233 terms from GO that were identified. Another caveat of this analysis was that we used proteins for which we had both turnover and abundance data. As a result, the majority of the proteins shown in Fig. 4B map to the mitochondria. To explore the cellular components in addition to mitochondria, we performed another analysis which is shown in Table 3. This analysis examined proteins individually in the abundance data set, for which we were not necessarily able to derive high-quality turnover information (due to low stable isotope incorporation in those proteins). Differential protein expression was defined based on the following criteria: p value <0.05, fold change >±1.5, matched peptides ≥2, and pooled quality control Coefficient of Variation <20%. At 120 h, there were 57 proteins that were differentially expressed between normal and microgravity (Table 3). Of the altered proteins in microgravity, 16 proteins increased in abundance while the remainder decreased in abundance. These proteins mapped to the mitochondria, the ER, and to the translational machinery. In line with the first analysis, greater than 50% of the proteins whose abundance increased were involved in mitochondrial processes, including mitochondrial protein homeostasis (mitochondrial protein import) and energy production. In contrast, a large percentage of the proteins whose abundance decreased were components of the ribosomal and endoplasmic reticulum machinery involved in cytoplasmic translation and co-translational protein folding. This reduction in protein involved in translation was consistent with the reduced protein turnover in *μg* relative to *1xg* found upon analysis of the rate of dynamic SILAC heavy amino acid incorporation in the proteome.

### ER stress and the UPR are occurring while mitochondrial integrity is preserved in microgravity

**Figure 5:**
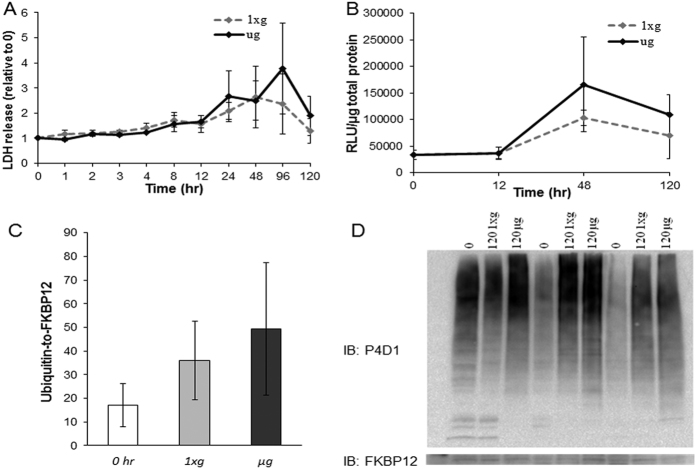
Surrogates of cell damage and protein degradation. (**A**) LDH release was not significantly different among groups (n = 4). (**B**) Caspase-3 activity was not different among groups (n = 3). (**C**) A bar chart representing the immunoblot of protein ubiquitination; there was no significant difference among groups. (**D**) Representative immunoblot of ubiquitinated proteins using antibody clone P4D1 (n = 3). LDH, lactate dehydrogenase; RLU, relative light unit.

Proteomics and gene set enrichment analyses indicate that microgravity differentially influences processes in the cytoplasm and mitochondria. Nonetheless, it is possible that microgravity exposure augments cell injury, contributing to the observed differences. To investigate this possibility the media from cells placed in *1xg* and *μg* conditions was examined for lactate dehydrogenase (LDH) levels at ten time points between 0 and 120 h (Fig. 5A). LDH is a soluble cytosolic enzyme, and its release is an indication of cell membrane permeability or damage. No statistically significant difference in LDH release from the cell was observed between *μg* and *1xg* at any time point, although LDH was elevated at 24 and 96 h compared to at 1 h within the *μg* group (p < 0.05, Repeated-measures ANOVA, n = 4; Fig. 5A). These data suggest that proteomic differences are not due to enhanced physical cell injury during microgravity exposure.

Another form of cell injury and death and also a surrogate measurement of mitochondrial integrity is apoptosis, which is indicated and executed by the protease caspase-3. We surveyed caspase-3 activity at 0, 12, 48, and 120 h in *μg* and *1xg* and did not observe a statistically significant difference in caspase-3 activity between *μg* and *1xg* at any time point (p < 0.05, Repeated-measures ANOVA, n = 3). There was an increase at 48 h compared to 0 and 12 h under both conditions, but this was not different between *μg* and *1xg* (Fig. 5B). This indicated that changes in the proteome between *μg* and *1xg* were not due to increased apoptosis. It additionally suggested that mitochondrial integrity was preserved in cardiomyocytes under the stress of microgravity.

Since protein ubiquitination is associated with proteins being tagged for degradation, we assessed global ubiquitination of protein lysates via western blot analysis at 0 h and from each group at 120 h. Again, no difference was observed between *μg* and *1xg* at 120 h or compared to 0 h (p = 0.21, one-way ANOVA, n = 3; Fig. 5C,D). Together, these data indicate that cardiomyocytes are not markedly damaged in response to simulated *μg* (LDH and Caspase-3 data), and that *μg* does not appear to enhance protein degradation (ubiquitination data), thus indicating that protein degradation is not playing a major role in the diminished protein turnover induced in simulated microgravity.

**Figure 6:**
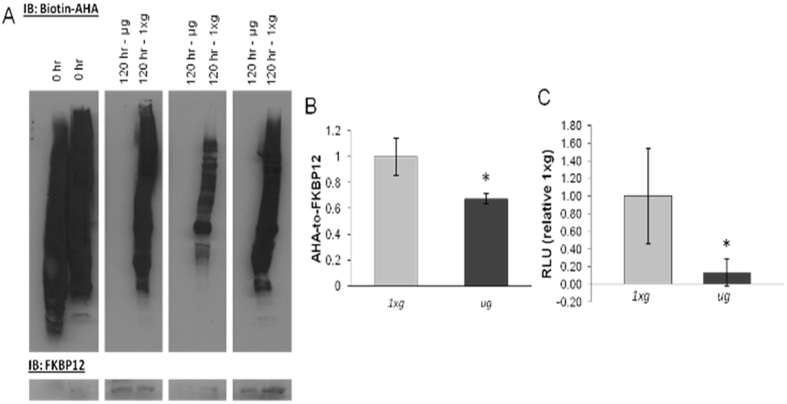
Translation is diminished in microgravity. (**A**) Immunoblot of biotin-tagged AHA, a methionine analog, and loading control FKBP12, together representing the decline in protein synthesis in *μg* at 120 h (n = 3, p = 0.003). AHA was added to the media for 2 hours at both 0 h and 120 h time points. (**B**) A bar chart representing the quantification of the densities of the AHA immunoblot. (**C**) Luciferase activity was markedly reduced in *μg* (n = 4, p = 0.02). AHA, azidohomoalanine; RLU, relative light unit.

To evaluate the influence of microgravity on protein synthesis, orthogonal experiments were performed utilizing the methionine analogue azidohomoalanine (AHA) which is incorporated into proteins via methionine tRNA and can be used to tag newly-translated proteins^20^. To measure protein synthesis across a small time window, cells were exchanged from Met- to AHA-containing media at 120 h, and labeling was performed for 2 h. Using the total protein lysates from each group, proteins were specifically tagged on the azido side chains with biotin and subsequently immunoblotted using HRP streptavidin (n = 3 per condition). AHA incorporation was robust after two hours of labeling, but was dramatically reduced at 120 h in *μg* relative to *1xg* (p = 0.003; Fig. 6A,B). These data demonstrate that protein synthesis, a factor contributing to overall protein turnover, is significantly diminished after cells have been exposed to *μg* for 120 h, compared to the same cells under normal gravity, and they suggest that the decreased protein turnover, as measured by RIA, reflected this decrease in protein synthesis.

To evaluate the consequences of *μg* on gene expression and translation from a non-chromosomal gene, the luciferase marker gene on an episome was introduced into NRCMs via an Adenoviral-luciferase vector 3 h prior to separating the cells into *μg* and *1xg* conditions. This allowed the luciferase gene to be transcribed and translated into protein using the cytoplasmic translational machinery. The adenovirus was removed when cells were placed into *μg* and *1xg* conditions at 0 h, and at 120 h, lysates from 120 h were evaluated for luciferase enzyme activity, which is a direct correlate to luciferase protein content since no post-translational modifications are necessary to initiate luciferase enzyme function^21^. Luminometry revealed that the content of luciferase protein in the cell was significantly diminished 7.5 fold in *μg* compared to *1xg* (p = 0.02, unpaired T-test, n = 4) as displayed in Fig. 6C. This was another indication that protein synthesis was reduced under the stress of microgravity, and further, it suggests that protein synthesis is not limited by chromosomal gene transcription, but rather due to translation.

## Discussion

Microgravity exposure is a known stressor that moderates mammalian physiology. For example, microgravity exposure has been reported to moderate cardiac atrophy^22^. Very little is known molecularly regarding the stress pathways activated or molecular processes leading to microgravity-induced cardiac atrophy. At the level of the proteome, there are three types of cellular stress responses: the classical “heat shock” response occurring in the cytoplasm; the unfolded protein response (UPR) occurring in the endoplasmic reticulum (UPR^ER^); and the UPR occurring in the mitochondria (UPR^MT^)^23^,^24^,^25^,^26^,^27^. The predominant response is dependent on the type of stress encountered, the cell type, the time point post-stress, and the cellular proliferation status.

Cells capable of cell division respond to stress by focusing resources for cellular division (i.e. entry into the cell cycle) rather than global protein synthesis, thereby increasing the chance of daughter cell survival. In contrast, specialized non-dividing cells, unable to re-enter the cell cycle, respond to stressful conditions through different mechanisms in efforts to conserve their functionality. A prime example are cardiomyocytes, the fundamental working unit of the heart. Under favorable conditions, cardiomyocytes maintain function by expending energy on the maintenance of the contractile machinery and the mitochondria. This maintenance is achieved through protein regulation, *i.e*. the balance of protein synthesis and protein degradation of the contractile apparatus and the mitochondria.

To gain a fundamental understanding of the microgravity stress response in non-dividing cells, we undertook a study looking at global proteomic changes in non-dividing cardiomyocytes in simulated microgravity. In addition, to understand rates of protein synthesis and stability in cardiomyocytes, we utilized a modification of dynamic SILAC. Dynamic SILAC typically is utilized in the reverse manner, where dividing cells are incubated for multiple divisions in heavy isotope-labeled media, which allows cellular amino acids to become fully heavy isotope-labeled. These cells then are exchanged into media containing native, non-isotope-labeled amino acids^13^,^16^, and the rate of protein synthesis and stability is followed by monitoring the rate of heavy isotope removal from the peptide population. Because neonatal rat cardiomyocytes are non-dividing cells and have a limited lifespan, they are incapable of being fully conditioned in heavy isotope-containing media. Thus, we performed dynamic SILAC in the forward direction^28^,^29^, starting with native isotope-labeled cells and switching the cells into heavy isotope-containing media. To our knowledge, this method of calculating protein turnover and simultaneous changes in overall protein expression by label-free quantitation has not been utilized^13^,^16^.

In non-dividing cardiomyocytes exposed to 120 h of simulated microgravity, levels of cytoplasmic heat shock proteins were diminished and protein ubiquitination was not different compared to normal gravity, suggesting that the classical heat shock response is not a predominant stress response to microgravity. In contrast, indicators of the UPR^ER^ and UPR^MT^ were increased. Overall, from quantitative and SILAC proteomics data and bioinformatics analysis, we observed that the content and processes of two cellular organelles were differentially affected by simulated microgravity, namely the mitochondria (up-regulated) and the cytoplasmic translational machinery, including the ribosomes and endoplasmic reticulum (down-regulated).

Mitochondria are the energy-producing powerhouses of the cell and compose >30% of cardiac cell volume, but they are exquisitely sensitive to stress-related damage^30^. They are constantly turning over through fusion and fission processes, being replaced with newer, more efficient mitochondria under optimal conditions^31^. Further, mitochondria may enhance their import machinery in response to stress^32^. Our data revealed that in response to simulated microgravity, two proteins directly involved in mitochondrial protein import were increased in abundance – mortalin and AFG3L2, which suggests there may be an increase in mitochondrial protein translation or a decrease in the degradation of these proteins under conditions of microgravity^33^, although we did not directly measure sub-compartmental, mitochondrial protein translation or degradation.

Mortalin, also known as mitochondrial stress 70 protein (mitoHsp-70) or grp75, is a mitochondrial chaperone protein located in the mitochondrial matrix and considered a major mediator of protein homeostasis contributing to mitochondrial biogenesis and energetics (reviewed in refs 34 and 35). Mortalin cooperates with Hsp60, another major chaperone protein in the mitochondrial matrix, to import unfolded proteins and subsequently fold the majority of the nascent synthesized proteins translocated into the mitochondria^35^. Mortalin is also found in several extra-mitochondrial sites, including the ER^36^, cytoplasmic vesicles, and the cytoplasm (reviewed in refs 34 and 35). In this extra-mitochondrial role, mortalin is a “pro-survival chaperone” that is anti-apoptotic. Moreover, it is one of the most important anti-apoptotic genes in that it protects the cell from many different stressors, including arsenite, glucose starvation, and ischemia-reperfusion.

AFG3L2 is involved in the same mitochondrial homeostasis pathway as mortalin^34^,^35^,^37^,^38^. However, as a member of the AAA ATPase family, AFG3L2 removes permanently damaged proteins that fail to refold and can only be rendered safe by proteolysis. In particular, AFG3L2 helps maintain mitochondrial protein quality control and degrades proteins of the respiratory chain that fail to assemble into the respiratory chain enzyme complexes^39^. Together, the up-regulation of mortalin and AFG3L2 suggest the mitochondria of cardiomyocytes respond to microgravity by supporting mitochondrial protein maintenance.

In contrast to the up-regulation of protein content and processes related to mitochondrial protein translation, proteins and processes mapping to translation occurring in the rough ER (RER) and ribosomes were down-regulated after 120 h of simulated microgravity exposure. Specifically, six ribosomal proteins were diminished in abundance −40 S components (S28, S9-like, S14-like), 60 S components (L30 like, L12), and ribosomal protein L4. Decreases in ribosomal proteins and tRNAs have been observed in skeletal muscle atrophy^40^,^41^. Also significantly decreased was asparaginyl-tRNA synthetase, an important component in translation occurring at the RER. Moreover, a mediator of RER and ribosome interaction, ribosome binding protein 1, was diminished in microgravity. Coupled to RER translation is import into and protein folding in the ER. Specific folding proteins that diminished in abundance in microgravity included translocon-associated protein subunit alpha precursor, reticulocalbin 3, reticulocalbin-1 precursor, and proteasome subunit beta type-2. Together, the down-regulation of these proteins suggests that protein translation via ribosomal translational machinery is reduced.

Both the ER and mitochondria are presently accepted as dynamic organelles capable of modifying their structure and function in response to changing environmental conditions. The ER and mitochondria interact both physiologically and functionally, and one of the most well-known and critical aspects of this interaction is calcium signaling between the two organelles^24^. However, another interaction between these organelles in cardiomyocytes appears to be a mechanism that biases mitochondrial protein translation over RER translation, most likely to maintain cellular energy production for global cell viability. Mortalin seems to be in two locations – between the ER and mitochondria and in the mitochondrial matrix – and may be the key molecule mediating this preferred translation.

The global down-regulation of protein translation was interpreted to be the ER unfolded protein response or UPR^ER^, of which a classic result is inhibition of translation. In combination with the reduction in translational proteins, the SILAC and AHA data further support that global cellular protein turnover and synthesis is significantly reduced in primary cardiac cells exposed to simulated microgravity. The SILAC data revealed decreased amino acid isotope incorporation, and that this reduction was not due to different amino acid availability or amino acid transport capability between gravity conditions. The AHA data depicted reductions at the translational level, since AHA is incorporated into protein via tRNA.

While there appears to be a global down-regulation in protein turnover as indicated by reduced RER translation and protein synthesis, protein turnover is also influenced by protein degradation; however, our data suggest there not to be an increase in protein degradation. Global ubiquitination was assessed, and although there was a qualitative increase in ubiquitination at 120 h compared to 0 h, no difference existed between *μg* and *1xg* at 120 h. Overall, the lack of change in LDH release, caspase-3 activity, or ubiquitination at 120 h between groups suggests there was minimal-to-no enhancement of cell damage and protein degradation between *μg* and *1xg*. Although, other contributors to degradation exist that were not evaluated, such as lysosomal proteases (e.g. cathepsins) and calpain activation, the surrogates used here provide evidence that simulated microgravity does not severely activate damage and degradation pathways in the cardiomyocyte. Lack of apoptosis induction observed in microgravity is consistent with other studies^42^,^43^. Further, mortalin, which was up-regulated, has been reported to be directly anti-apoptotic^44^.

Cytoarchitecture is linked to mitochondrial localization and energetics^30^, whereby the cytoarchitecture comprising structural and contractile proteins may moderate energetic micro domains and energetic cross talk. The abundance of seven structural and/or contractile proteins was significantly diminished in microgravity. Notably, myosin regulatory light chain and tropomyosin (Table 1) diminishment is a hallmark characteristic of atrophy^41^. How potential microgravity-moderated cytoarchitectural changes may mediate mitochondrial localization and energetics deserves further research.

Cardiac atrophy is a phenomenon that can lead to orthostatic intolerance and has been observed in spaceflight studies with rats, human bed rest studies, and is identified as a major challenge for crew returning from long duration missions^45^,^46^,^47^. However, the mechanisms leading to this phenotype are unclear. Our data suggest that under exposure to microgravity, cardiac cells respond by upregulating mitochondrial proteins to preserve energy production and promote short-term survival, but are not able to overcome apparent disruption of protein translation at the RER because of decreases in the levels of specific ribosomal proteins. This disruption at the RER ultimately decreases overall protein translation and contributes to the development of cardiac atrophy.

Our data suggest that a certain degree of protein misfolding, possibly compartmentalized misfolding is occurring in microgravity. The trigger for this remains unclear. Stress responses generally originate at the cell surface, trigger signal transduction cascades which induce induction of nuclear gene transcription; however, microgravity affects the entire cell equally and at once, so this may represent an entirely new type of cellular stress response. Mortalin may possibly be a key regulator. The upregulation of mortalin may be a means of selectively allowing continuing mitochondrial translation at the expense of RER translation so that the energetics of the cell are maintained. Understanding the interplay of mortalin in pathways and biological processes will provide a better understanding of the coordinated cellular response to microgravity, and further studies to improve understanding of the cellular responses to microgravity will help us prepare for extended travel beyond Earth’s orbit.

## Materials and Methods

### Neonatal Rat Cardiomyocyte (NRCM) isolation

Animal experiments were approved by Duke University Medical Center Institutional Animal Care and Use Committee (IACUC) and are in accordance with United States federal and North Carolina state regulations. NRCMs were isolated from ventricles of 2-day-old Sprague-Dawley rats (Charles River Laboratories) as previously reported^48^.

### Experimental design including dynamic SILAC labeling of NRCM

Following isolation, NRCMs were plated on 100 mm tissue culture dishes. Cells were collected by trypsinization, exchanged into DMEM:F12 SILAC media (Thermo Scientific; prepared as previously described^49^, and seeded onto Cytodex-3 microcarrier beads (Sigma). Normal gravity (*1xg*) cells were plated on tissue culture dishes containing 10 ml SILAC media. Simulated *μg* cells were transferred to rotating wall vessels (RWV; Synthecon, Inc., Houston, TX) containing 10 ml SILAC media. Initially, the cell-bead mixture was allowed to sit in the RWV for 2 h to enhance cell attachment prior to rotation. Both the *1xg* and *μg* cells were cultured for 12, 48, and 120 h, with fresh media added every 48 h. NRCMs were collected by trypsinization, washed three times in 50 mM ammonium bicarbonate (pH 8.0), pelleted (300 xg for 2 min), flash frozen in liquid N_2_, and stored at −80 °C until analysis.

### Sample Preparation and Protein Isolation

Immediately upon removal from −80 °C storage, cell pellets were suspended in 100 μL of 0.25% RapiGest (Waters Corp.) in 50 mM ammonium bicarbonate, pH 8.0. This volume was pipetted up and down several times to lyse the cell pellet, and then each was transferred to a 1.5 mL Eppendorf low-bind tube. An additional 100 μL of 0.25% RapiGest was added to each conical tube as a rinse and added to the first 100 μL aliquot. Tubes were then re-frozen at −80 °C for 10 min, removed from freezer, and thawed on ice. This freeze/thaw cycle was repeated. Each sample was probe sonicated for 3 cycles of 5 s bursts at power level 3, cooling on ice between bursts. Samples were centrifuged at 15,000 rpm for 5 min, and a very small pellet was visible at the bottom of each tube. Protein concentrations were determined by mini-Bradford assay (BioRad, Inc.) against a bovine serum albumin (BSA) calibration curve. 25 μg from each sample was removed (volumes based on concentration), and added to new 0.5 μL tubes. Then each sample was normalized to 86.2 μL total with 0.25% RapiGest (based on the sample with the lowest concentration). Samples were then digested according to the in-solution tryptic digestion protocol detailed here: http://www.genome.duke.edu/cores/proteomics/sample-preparation/. Briefly, samples were reduced to 10 mM dithiothreitol final for 15 min at 80 °C while shaking and alkylated to 20 mM iodoacetamide final for 30 min at room temperature in the dark. Sequencing grade porcine trypsin was added at 1:50 enzyme: protein ratio and samples were incubated at 37 °C overnight while shaking. Following overnight digestion, RapiGest surfactant was hydrolyzed by addition of 1% TFA/2% ACN final. All samples were also spiked with ADH1_YEAST digest (Massprep standard, Waters Corp.) as a surrogate standard (50 fmol ADH per μg total protein). All samples were heated at 60 °C for 2 h and centrifuged at 15,000 rpm for 10 min before pipetting all supernatant into individual TotalRecovery LC vials (Waters, Corp.). 3 μL was pipetted from each and combined to make a pool for QC purposes.

### Data collection for LC-MS/MS proteomics

All samples were randomized within a biological replicate group for acquisition order. Run order for the raw data collection is displayed in S1. Before each replicate group queue, 1 μg from the pooled sample was injected, and was also injected 4× at the end of the queue, for a total of 7 QC pool runs. One of the seven pooled analyses was run in data-dependent acquisition mode (DDA) for complementary peptide identifications whereas the others were performed as follows.

Quantitative LC/MS/MS was performed on 1 μg of protein digest per sample, using a nanoAcquity UPLC system (Waters Corp) coupled to a Synapt G2 HDMS high resolution accurate mass tandem mass spectrometer (Waters Corp.) via a nanoelectrospray ionization source. Briefly, the sample was first trapped on a Symmetry C18 300 mm × 180 mm trapping column (5 μl/min at 99.9/0.1 v/v water/acetonitrile), after which the analytical separation was performed using a 1.7 μm Acquity BEH130 C18 75 mm × 250 mm column (Waters Corp.) using a 90-min gradient of 5 to 40% acetonitrile with 0.1% formic acid at a flow rate of 400 nanoliters/minute (nL/min) with a column temperature of 55 °C. Data collection on the Synapt G2 mass spectrometer was performed in ion-mobility assisted data-independent acquisition (HDDIA or HDMSE) mode, using 0.6 s alternating cycle time between low (6 V) and high (27–50 V) collision energy (CE). Scans performed at low CE measure peptide accurate mass and intensity (abundance), while scans at elevated CE allow for qualitative identification of the resulting peptide fragments via database searching. The total analysis cycle time for each sample injection was approximately 1.5 h.

Following the 28 analyses (with the additional QC standard injections), data was imported into Rosetta Elucidator v3.3 (Rosetta Biosoftware, Inc.), and all LC-MS files were aligned based on the accurate mass and retention time of detected ions (“features”) using PeakTeller algorithm (Elucidator). The relative peptide abundance was calculated based on area-under-the-curve (AUC) of aligned features across all runs. The dataset had 280,291 quantified features and 24,433 annotated spectra. This MS/MS data was searched against a NCBI RefSeq database with *rattus norvegicus* taxonomy (25,485 forward entries) appended with a decoy reverse-sequence of each forward entry for false positive rate determination. The sequences for ADH_yeast, CASA1_bovin, and CASA2_bovin were also appended to the database representing internal standards used. Static mass modification corresponding to carbamidomethylation (from alkylation protocol) was required on Cys residues, whereas dynamic mass modifications corresponding to oxidation was allowed on Met residues, deamidation was allowed on Asn and Gln residues, and heavy labeled (^13^C^15^N) Arg and Lys. After individual peptide scoring using PeptideProphet algorithm (Elucidator), the data was annotated at a <1% peptide false discovery rate. This analysis yielded identifications for 6,339 peptides and 864 proteins across samples, including 493 proteins with two or more peptides quantified. For quantitative processing, only 25 of the 28 runs were used (only the 4 pooled analyses performed in the exact same way were used whereas the last 3 were excluded from quantitation). The data was first curated to contain only high-quality peptides with appropriate chromatographic peak shape, and the dataset was intensity scaled to the robust median across all samples analyzed; the final quantitative dataset was based on 6,174 peptides and contained 848 proteins.

### Proteomic statistical analyses

For analysis of relative protein expression between conditions (time or microgravity) both heavy- and native isotope-labeled peptides were quantified by accurate mass and retention time alignment, and the heavy and light forms of each peptides were summed to yield a total expression value for each peptide. Peptide intensities were summed to the protein level to obtain values for each protein in each sample analyzed, and protein intensities were log 2 transformed prior to statistical analysis. The criteria for significance are noted in Results. Protein expression data were z-score normalized. Protein-level expression data were analyzed after log 2 transformation followed by Error-Weighted Analysis of Variance (ANOVA) with Bonferroni correction to control for multiple-hypothesis testing. Tables for peptide expression (S2 and for protein expression (S3) are displayed in Supplemental Information. PCA was performed using the mass spec protein expression intensity values from Table S3. The PCA plot was made using FactoMineR^50^. Ellipses represent 99.999% confidence intervals around each of the time point groups. The smallest variability among any sample group is among 0 h (in blue) and 12 h (short dashed) samples, followed by 48 h (long dashed) and 120 h (solid line). The QC pool samples (in red) are nearly indistinguishable in the center of the PCA, showing technical variability is far less than biological variability.

### Protein turnover measurements

Protein turnover was determined by dynamic SILAC, similar to previously described^13^,^16^. Selection of peptides for use in this analysis required several criteria: (1) heavy and light forms of the peptide were independently identified by MS/MS; (2) the retention time of heavy and light peptides could not differ by more than 0.5 min; (3) The noise of the heavy-isotope peak must be less than 1/10 of the light isotope peak at T = 0, which selected peaks with S/N of 10:1 or more. The relative isotope abundance (RIA) was calculated as in Equation 2 using the method of Doherty *et al*.^16^. These calculations assume that the amino acid pool contains 100% heavy isotope-labeled Lys and Arg; else, this will result in incomplete labeling at max time, and RIA (∞) <1. The RIA, if different from 1, should also be the same in *μg* and *1xg*. Dynamic SILAC incorporation measures for a subset of 359 curated proteins (S4) are displayed in Supplemental Information.

### Bioinformatics Analysis

#### Data Preparation

For the gene enrichment/pathways analysis 478 peptides were chosen from the protein abundance data set using the criterion of matched peptides ≥ 2, and pooled quality control Coefficient of Variation <20%. In addition, there were 359 peptides for which turnover information was available from the SILAC component of the experiment. The proteomics data was transformed into gene-space to facilitate functional enrichment analysis. To transform this data, protein RefSeq identifiers were mapped to gene symbols using Bioconductor’s MyGene package^18^. For the protein abundance data set, the 478 RefSeq identifiers were mapped to 474 distinct gene symbols. Similarly, the 359 RefSeq identifiers in the SILAC data were mapped to 195 gene symbols.

Approximately 3,000 gene functional annotations were collected, comprising Gene Ontology (GO) biological processes, molecular functions, cellular components and canonical pathways from KEGG, BIOCARTA, REACTOME, and Pathway Interaction Database (PID). We used a Gene Set Enrichment Analysis technique^51^ to identify statistically up- and down-regulated gene set in the protein abundance and turnover data sets (Tables 1 and 2).

### Assays of cell death and protein ubiquitination

LDH in culture media was detected using the CytoTox-ONE Homogenous Membrane Integrity Assay (Promega). Caspase-3 activity was performed on cell lysates using Caspase-GLO 3/7 assay (Promega) as previously described^48^. Protein ubiquitination was quantified by western blotting using P4D1 (Santa Cruz sc-8017).

### Assays for protein turnover

Cells were starved of Met and treated with azidohomoalanine (AHA) as previously described^52^. AHA labeling was performed for 2 h at 118–120 h. AHA incorporation in cell lysates was assessed by reaction with sulfo-dibenzocyclooctyne-biotin (Sigma) followed by western blotting. Coomassie staining was used to confirm protein loading. Membranes were stripped and reprobed for FKBP12 (ab108420, abcam, Cambridge, MA) as a loading control. AHA incorporation was quantified by densitometry using Image J (NIH, Bethesda, MD) after normalization to FKPB12. Adenovirus was used to express luciferase in NRCMs. Cells were treated with Ad-luciferase (multiplicity of infection of 100 particles per cell) for 3 h prior to group separation. At 120 h, cell pellets were assayed for luciferase activity as described previously^48^.

### Assay statistical analyses

All analyses independent from open platform proteomics were performed using JMP v11 (SAS, Cary, NC). Statistical significance was set at p = 0.05.

## Additional Information

**How to cite this article**: Feger, B. J. *et al*. Microgravity induces proteomics changes involved in endoplasmic reticulum stress and mitochondrial protection. *Sci. Rep*. **6**, 34091; doi: 10.1038/srep34091 (2016).

## Supplementary Material

Supplementary Information

Supplementary Tables

## Figures and Tables

**Table 1:** Gene Set Enrichment Analysis for Protein Abundance Data.

UP-REGULATED	FDR
METABOLIC	
CARBOXYLIC_ACID_METABOLIC_PROCESS	3.36E-002
FATTY_ACID_METABOLIC_PROCESS	3.11E-002
KEGG_FATTY_ACID_METABOLISM	1.40E-002
MONOCARBOXYLIC_ACID_METABOLIC_PROCESS	3.07E-002
ORGANIC_ACID_METABOLIC_PROCESS	3.48E-002
REACTOME_FATTY_ACID_TRIACYLGLYCEROL_AND_KETONE_BODY_METABOLISM	3.80E-002
REACTOME_METABOLISM_OF_AMINO_ACIDS_AND_DERIVATIVES	4.85E-002
REACTOME_METABOLISM_OF_LIPIDS_AND_LIPOPROTEINS	3.45E-002
REACTOME_PYRUVATE_METABOLISM_AND_CITRIC_ACID_TCA_CYCLE	6.93E-004
MITOCHONDRION	
MITOCHONDRIAL_LUMEN	3.74E-002
MITOCHONDRIAL_MATRIX	3.91E-002
MITOCHONDRIAL_MEMBRANE	4.68E-002
MITOCHONDRIAL_PART	3.57E-003
MITOCHONDRION	0.00E + 000
ELECTRON TRANSPORT	
REACTOME_RESPIRATORY_ELECTRON_TRANSPORT	4.69E-002
REACTOME_RESPIRATORY_ELECTRON_TRANSPORT_ATP_SYNTHESIS_BY_CHEMIOSMOTIC_COUPLING_AND_HEAT_PRODUCTION_BY_UNCOUPLING_PROTEINS_	3.19E-002
REACTOME_TCA_CYCLE_AND_RESPIRATORY_ELECTRON_TRANSPORT	2.61E-004
CYTOPLASMIC_PART	3.71E-002
KEGG_CITRATE_CYCLE_TCA_CYCLE	3.45E-002
REACTOME_CITRIC_ACID_CYCLE_TCA_CYCLE	3.62E-004
KEGG_VALINE_LEUCINE_AND_ISOLEUCINE_DEGRADATION	9.91E-003
OXIDOREDUCTASE_ACTIVITY_ACTING_ON_NADH_OR_NADPH	3.61E-002
DISEASES	
KEGG_ALZHEIMERS_DISEASE	3.29E-002
KEGG_HUNTINGTONS_DISEASE	1.74E-002
KEGG_PARKINSONS_DISEASE	3.33E-002
DOWN-REGULATED	
KEGG_RIBOSOME	3.98E-002
REACTOME_INFLUENZA_LIFE_CYCLE	4.50E-002
REACTOME_INFLUENZA_VIRAL_RNA_TRANSCRIPTION_AND_REPLICATION	4.48E-002
REACTOME_SRP_DEPENDENT_COTRANSLATIONAL_PROTEIN_TARGETING_TO_MEMBRANE	2.50E-002

**Table 2:** Gene Set Enrichment Analysis for Protein Turnover Data.

UP-REGULATED	FDR
MITOCHONDRION	
MITOCHONDRIAL_ENVELOPE	4.78E-004
MITOCHONDRIAL_INNER_MEMBRANE	3.65E-003
MITOCHONDRIAL_MEMBRANE	1.12E-003
MITOCHONDRIAL_MEMBRANE_PART	5.26E-002
MITOCHONDRIAL_PART	8.56E-005
MITOCHONDRION	0.00E + 000
ENVELOPE	3.63E-003
ORGANELLE_ENVELOPE	4.13E-003
ORGANELLE_INNER_MEMBRANE	3.49E-003
REGULATION_OF_MULTICELLULAR_ORGANISMAL_PROCESS	4.45E-003
ELECTRON TRANSPORT	
ACTIVE_TRANSMEMBRANE_TRANSPORTER_ACTIVITY	2.79E-002
ELECTRON_TRANSPORT_GO_0006118	9.26E-002
ION_TRANSMEMBRANE_TRANSPORTER_ACTIVITY	1.54E-002
REACTOME_RESPIRATORY_ELECTRON_TRANSPORT	3.28E-002
REACTOME_RESPIRATORY_ELECTRON_TRANSPORT_ATP_SYNTHESIS_BY_CHEMIOSMOTIC_COUPLING_AND_HEAT_PRODUCTION_BY_UNCOUPLING_PROTEINS_	1.87E-003
REACTOME_TCA_CYCLE_AND_RESPIRATORY_ELECTRON_TRANSPORT	6.48E-005
REACTOME_TRANSMEMBRANE_TRANSPORT_OF_SMALL_MOLECULES	8.61E-002
KEGG_CITRATE_CYCLE_TCA_CYCLE	5.89E-003
REACTOME_CITRIC_ACID_CYCLE_TCA_CYCLE	6.47E-002
REACTOME_FORMATION_OF_ATP_BY_CHEMIOSMOTIC_COUPLING	8.66E-002
KEGG_OXIDATIVE_PHOSPHORYLATION	3.95E-003
DISEASES	
KEGG_ALZHEIMERS_DISEASE	8.00E-003
KEGG_HUNTINGTONS_DISEASE	9.80E-004
KEGG_PARKINSONS_DISEASE	5.81E-003
MUSCLE DEVELOPMENT AND CONTRACTION	
KEGG_CARDIAC_MUSCLE_CONTRACTION	4.50E-003
HEART_DEVELOPMENT	1.04E-002
MUSCLE_DEVELOPMENT	1.05E-002
REGULATION_OF_HEART_CONTRACTION	5.16E-002
DOWN-REGULATED	
ESTABLISHMENT_OF_PROTEIN_LOCALIZATION	6.68E-002
MACROMOLECULE_LOCALIZATION	7.70E-002
PROTEIN_LOCALIZATION	8.78E-002

**Table 3:** Individual protein abundance alterations in microgravity.

Primary Protein Name	Protein Description	%CV	FC @ 120 h (*ug* v *1xg*)	Function
392351353	Myosin-13	9.9	3.5	Molecular motor
11693154	Platelet-activating factor acetylhydrolase IB subunit beta	11.5	2.7	Activity of RhoGTPases, actin polymerization
198442897	AFG3-like protein 2	10.3	2.5	Mitochondrial protein homeostasis
9507135	Spectrin beta chain, brain 2	5.2	2.1	Actin binding
293359790	Protein FAM179A-like isoform 1	3.6	1.9	Unknown function
62945328	Protein NipSnap homolog 2	11.9	1.8	Mitochondria, neg regulation of ATP citrate synthase activity
25742739	Long-chain-fatty-acid–CoA ligase 1	2.5	1.7	Activates breakdown of complex FA
48675862	Acyl-coenzyme A thioesterase 2, mitochondrial	2.0	1.7	Catalyzes hydrolysis of acyl-CoA to free FA and co enzyme A
392347468	Poly (rC)-binding protein 1	6.2	1.6	mrna import to mitochondria
56605722	Serine hydroxymethyltransferase, mitochondrial	6.2	1.6	Conversion l-serine to glycine, provides 1 carbon units to cell
113205496	Pyruvate dehydrogenase complex, component X	2.1	1.6	Mitochondrial, tether E3 dimers to E2 core
392341350	Glyceraldehyde-3-phosphate dehydrogenase-like	1.3	1.5	Breaks down glucose for energy and carbon
13994225	3-hydroxyacyl-CoA dehydrogenase type-2	8.6	1.5	Mitochondrial tRNA maturation
11968102	Ornithine aminotransferase, mitochondrial precursor	5.2	1.5	Processes excess nitrogen during protein breakdown
392351018	Sarcalumenin	3.2	1.5	Calcium buffering in SR
410110929	Stress-70 protein, mitochondrial	3.1	1.5	Mitochondrial protein homeostasis
209954804	Plastin-3	9.5	−24.3	EF-hand protein, bone?
293346882	Mitochondrial aspartate aminotransferase-like	18.0	−22.2	metabolite exchange between mitochondria and cytosol, uptake of long chain free fatty acids
8394079	Proteasome subunit beta type-2	11.8	−15.4	ATP dependent proteolytic activity
157818179	Elongation factor 1-beta	5.3	−13.4	Translation elongation, gdp to gtp exchange
157787127	40 S ribosomal protein S28	8.2	−12.6	Ribosomal protein, translation
392348740	Laminin subunit beta-1	4.2	−12.2	ECM structural component
77993298	Translocon-associated protein subunit alpha precursor	5.3	−12.0	ER protein, ER UPR
12018252	Transketolase	14.2	−8.9	Pentose phosphate pathway
56744249	Reticulocalbin 3, EF-hand calcium binding domain precursor	4.9	−7.6	ER Calcium binding
40254781	Rab GDP dissociation inhibitor beta	15.7	−6.8	Regulates gdp/gtp exchange of most rab proteins, poly A RNA binding
392342369	60 S ribosomal protein L30-like	3.9	−6.7	Ribosomal protein, translation
6981326	Protein S100-A4	7.0	−6.5	Ca binding, polyA RNA binding
61556832	Adenine phosphoribosyltransferase	13.5	−6.3	AMP biosynthesis salvage pathway
6981574	SPARC precursor	15.7	−6.2	Regulates cell growth, thru ECM and binds calcium
27665858	40 S ribosomal protein S9-like	12.2	−5.9	Ribosomal structural protein
392346755	Ribosome-binding protein 1	14.7	−5.6	Mediates interaction between ribosome and ER
392347136	Collagen alpha-2(I) chain-like isoform 1	7.2	−5.5	Extracellular matrix
13592133	Actin, cytoplasmic 1	3.7	−5.3	motility
203097140	Myosin regulatory light chain RLC-A	9.0	−5.2	Muscle contraction
392348865	Histone H2A. V-like	4.5	−4.7	Chromosomal binding
77404180	Ras-related protein Rab-4A	12.5	−4.6	ATPase activator activity
157819753	Reticulocalbin-1 precursor	13.3	−4.5	ER calcium binding
61556967	Elongation factor 1-delta	9.3	−4.1	Transfer of aminoacyl-tRNA to ribosome
6981672	Tropomyosin alpha-4 chain	13.0	−3.7	Muscle contraction
6978589	Non-muscle caldesmon	5.7	−3.6	Muscle contraction
157822227	60 S ribosomal protein L12	7.8	−3.4	translation
148747365	Heat shock protein HSP 90-beta	2.3	−3.4	Classic heat shock protein
392356007	Actin, cytoplasmic 1-like	5.0	−3.3	Cell motility
9506845	Rab GTPase-binding effector protein 1	15.1	−3.2	Membrane trafficking, protein localization
157786744	Dihydropyrimidinase-related protein 2	4.5	−3.0	Cytoskeletal organization
6981240	Myosin light chain 3	4.3	−2.5	Muscle contraction
124107592	Unconventional myosin-Ic	6.1	−2.3	Associated with transcriptionally active ribosomal genes
12083607	40 S ribosomal protein S14-like	2.5	−2.2	translation
56090293	Pyruvate dehydrogenase E1 component subunit beta, mitochondrial precursor	4.1	−2.1	metabolism
219275589	Asparaginyl-tRNA synthetase, cytoplasmic isoform 2	3.5	−2.1	translation
293350511	M2 pyruvate kinase-like isoform 1	2.1	−2.0	metabolism
155369650	Myosin light polypeptide 6	6.2	−1.8	Muscle contraction
11968086	Ribosomal protein L4	5.1	−1.7	translation
66730475	Tropomyosin beta chain	7.2	−1.6	Muscle contraction
6981236	Myosin-9	4.8	−1.6	Cytoskeletal organization
6981666	Troponin T, cardiac muscle	3.5	−1.5	Cardiac structure
