# Supplementary material for: Microgravity induces proteomics changes involved in endoplasmic reticulum stress and mitochondrial protection
Source: Sci Rep. 2016 Sep 27;6:34091. doi: 10.1038/srep34091 (PMC5037457; doi:10.1038/srep34091)
Supplement: Supplementary Information [file srep34091-s1.pdf]

Title: Microgravity induces proteomics changes involved in endoplasmic reticulum stress and mitochondrial protection

Author affiliation:

Bryan J. Feger [a, 1], J. Will Thompson [b,1], Laura G. Dubois [b], Reddy P. Kommaddi [c], Matthew W. Foster [b,c], Rajashree Mishra [a], Sudha K. Shenoy [c], Yoichiro Shibata [d], Yared H. Kidane [e, f], M. Arthur Moseley [b], Lisa S Carnell [g], and Dawn E. Bowles [a *]

[a] Department of Surgery, Duke University Medical Center, Durham, NC 27710, USA

[b] Duke Proteomics and Metabolomics Shared Resource, Duke University Medical Center, Durham, NC 27710, USA

[c] Department of Medicine, Duke University Medical Center, Durham, NC 27710, USA

[d] Department of Genetics, the Carolina Center for Genome Sciences, and the Lineberger Comprehensive Cancer Center, University of North Carolina, Chapel Hill, NC 27599

[e] Wyle Science, Technology and Engineering Group, Houston, TX 77058, USA

[f] NASA Johnson Space Center, Houston, TX 77058, USA

[g] NASA Langley Research Center, Hampton, VA, 23666, USA

[1]B.J.F and J.W.T contributed equally to this work.

***Corresponding author:**

Dawn E. Bowles

DUMC 2642

MSRB1 Rm 401B

Research Drive

Durham, NC 27710, USA

Telephone: 919-668-1947

Email: dawn.bowles@duke.edu

Keywords: gravity, microgravity, cellular stress, dynamic SILAC, proteomics, protein synthesis, spaceflight, atrophy, protein turnover, ribosomal proteins, mitochondrial homeostasis, mass spectrometry

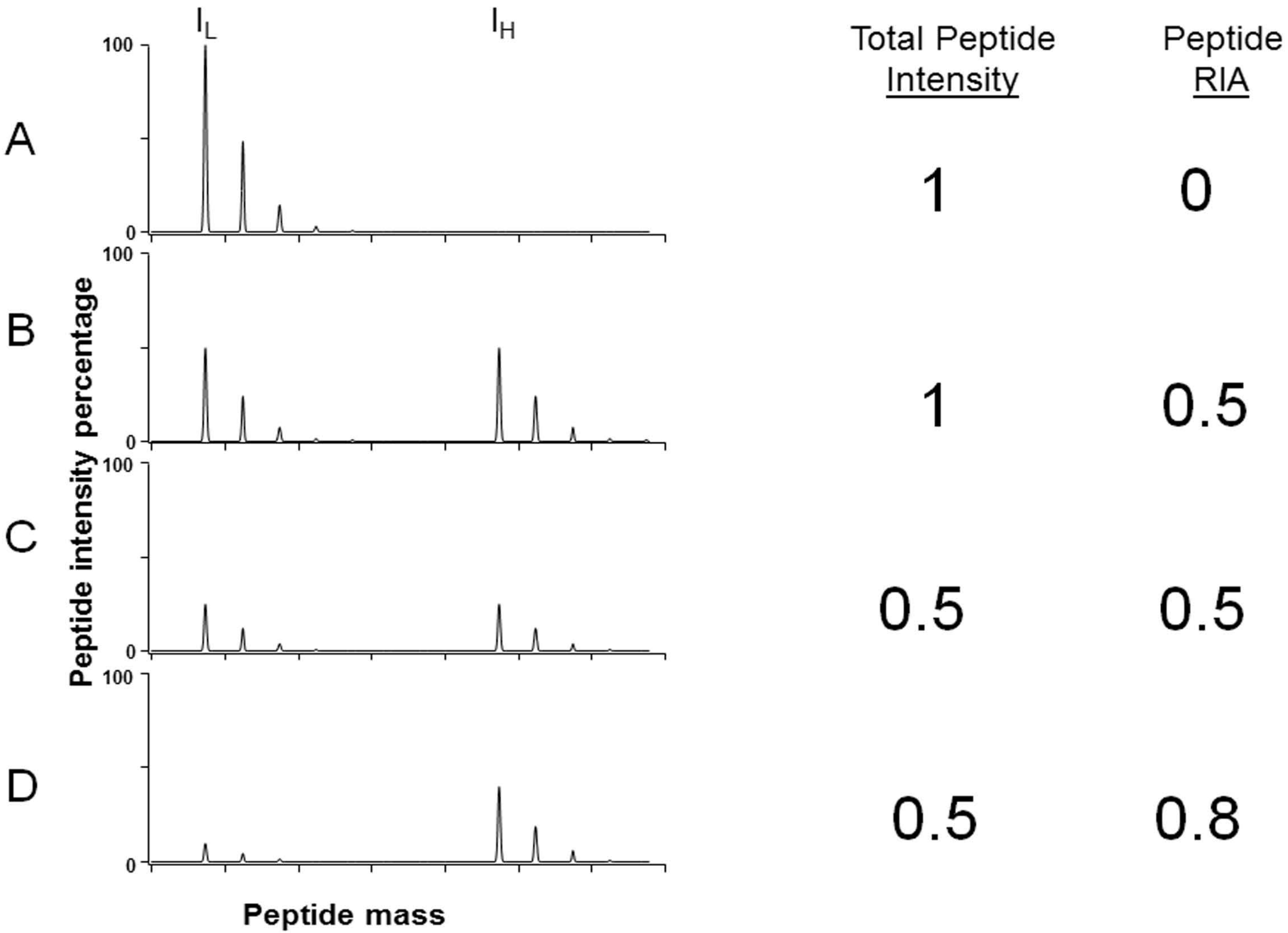

I_L
I_H
Total Peptide Intensity
Peptide RIA
A
1
0
B
1
0.5
C
0.5
0.5
D
0.5
0.8
100
0
Peptide intensity percentage
Peptide mass

$$\text{Total Peptide Intensity} = I_L + I_H \qquad \text{(Equation 1)}$$

$$\text{Relative Isotope Abundance (RIA)} = \frac{I_H}{I_L + I_H} \qquad \text{(Equation 2)}$$

Supplemental Figure legends

Supplemental Figure 1. Theoretical examples of utilizing stable-isotope incorporation and label-free quantitation to simultaneously determine relative peptide intensity and relative isotope abundance (RIA). Four different pairs of heavy/light peptides show four different hypothetical states of a system (A-D). Calculations of the relative abundance and the Peptide RIA using Equations 1 and 2 (Figure 4), respectively. State B shows no change in abundance relative to State A, but 50% isotope incorporation (RIA = 0.5). States C and D show a 50% reduction in peptide abundance compared to States A and B, and also show RIA of 0.5 and 0.8, respectively.

Supplemental Figure 2. Equation 1 was used to determine total peptide abundance; Equation 2 was used to determine peptide RIA. I_L, light isotope; I_H, heavy isotope.
